# Supplementary material for: Complexome profiling of the Chlamydomonas psb28 mutant reveals TEF5 as an early PSII assembly factor
Source: Plant Cell. 2025 Mar 17;37(6):koaf055. doi: 10.1093/plcell/koaf055 (PMC12164590; doi:10.1093/plcell/koaf055)
Supplement: koaf055_Supplementary_Data [file koaf055_supplementary_data.zip › psb28_interactive.zip!/plots/intensity/qualityPlots/peptides/PSBQ_Cre08.g372450.t1.2.html]

### Cre08.g372450.t1.2

Trivial name: PSBQ  
SeqCoverage [median]: 54.30  
Mass: 21.82 kDa  
  
 

| Sequence | MissedCleavage | Mass [Da] | wtIntensity | muIntensity | Score |
| --- | --- | --- | --- | --- | --- |
| AALGLRK | 1 | 727.47 | 142233.80 | 23575.41 | 155.81 |
| AKLDSVLAAVL | 1 | 1098.66 | 479992.44 | 302622.22 | 128.00 |
| ASLDETK | 0 | 762.38 | 869115.36 | 127000.92 | 200.12 |
| ASLDETKK | 1 | 890.47 | 10948.20 | 0.00 | 127.46 |
| ASLDETKKR | 2 | 1046.57 | 36046.09 | 0.00 | 131.41 |
| DLDLPQNVR | 0 | 1068.56 | 2023080.28 | 608997.13 | 223.83 |
| DLDLPQNVREGFTQAR | 1 | 1857.93 | 226565.20 | 44866.84 | 159.34 |
| DRGFDLIYEAR | 1 | 1353.67 | 649251.82 | 360352.16 | 140.00 |
| EGFTQAR | 0 | 807.39 | 757229.28 | 261370.87 | 182.95 |
| FDLNTLASTK | 0 | 1108.58 | 1519988.17 | 744283.11 | 262.48 |
| FDLNTLASTKEK | 1 | 1365.71 | 67422.95 | 22943.76 | 145.81 |
| FDLNTLASTKEKEAK | 2 | 1693.89 | 11740.37 | 13499.16 | 192.32 |
| FDLNTLASTKEKEAKK | 3 | 1821.98 | 2264.08 | 0.00 | 87.14 |
| GFDLIYEAR | 0 | 1082.54 | 649976.46 | 347032.97 | 220.51 |
| IDADLDVFIQK | 0 | 1275.67 | 54038.73 | 75297.59 | 264.90 |
| KAALGLR | 1 | 727.47 | 520010.30 | 89987.98 | 170.31 |
| KAALGLRK | 2 | 855.57 | 3184.61 | 0.00 | 140.12 |
| KLEITK | 1 | 730.46 | 914475.26 | 287295.78 | 190.49 |
| LDSVLAAVL | 0 | 899.53 | 22144.93 | 0.00 | 138.48 |
| QVGTLRFDLNTLASTK | 1 | 1762.96 | 40922.23 | 4355.41 | 113.47 |
| QVGTLRFDLNTLASTKEK | 2 | 2020.10 | 15237.70 | 2292.52 | 169.21 |
| QVGTLRFDLNTLASTKEKEAK | 3 | 2348.27 | 1717.70 | 0.00 | 31.36 |
| RQVGTLR | 1 | 828.49 | 53423.57 | 17219.21 | 158.36 |
| RQVGTLRFDLNTLASTK | 2 | 1919.06 | 13721.90 | 840.19 | 77.56 |
| SVRDRGFDLIYEAR | 2 | 1695.87 | 19787.37 | 0.00 | 142.85 |
| SYWTEAR | 0 | 911.41 | 386026.25 | 28231.93 | 206.65 |
| SYWTEAREQLR | 1 | 1437.70 | 2897.17 | 0.00 | 62.30 |
| SYWTEAREQLRR | 2 | 1593.80 | 4007.28 | 2968.22 | 88.44 |
|  |  |  | 9497449.5 | 3365033.4 |  |
